# Supplementary material for: Extreme parsimony in ATP consumption by 20S complexes in the global disassembly of single SNARE complexes
Source: Nat Commun. 2021 May 28;12:3206. doi: 10.1038/s41467-021-23530-0 (PMC8163800; doi:10.1038/s41467-021-23530-0)
Supplement: Supplementary file 1 — Supplementary Information [file 41467_2021_23530_MOESM1_ESM.pdf]

# Extreme parsimony in ATP consumption by 20S complexes in the global disassembly of single SNARE complexes

Changwon Kim[1,7], Min Ju Shon[1,2,7], Sung Hyun Kim[1,6], Gee Sung Eun[1], Je-Kyung Ryu[3,6], Changbong Hyeon[4], Reinhard Jahn[5] and Tae-Young Yoon[1,*]

[1]School of Biological Sciences and Institute for Molecular Biology and Genetics, Seoul National University, Seoul 08826, South Korea

[2]Department of Physics, Pohang University of Science and Technology, Pohang, Gyeongbuk 37673, South Korea

[3]Department of Physics, KAIST, Daejeon 305-701, South Korea

[4]Korea Institute for Advanced Study, Seoul 02455, South Korea

[5]Department of Neurobiology, Max-Planck-Institute for Biophysical Chemistry, 37077 Göttingen, Germany

[6]Present address: Department of Bionanoscience, Kavli Institute of Technology, Delft University of Technology, the Netherlands

[7]These authors equally contributed to this work.

*Correspondence: tyyoon@snu.ac.kr.

Content:

Supplementary Figs. 1-8

Supplementary References

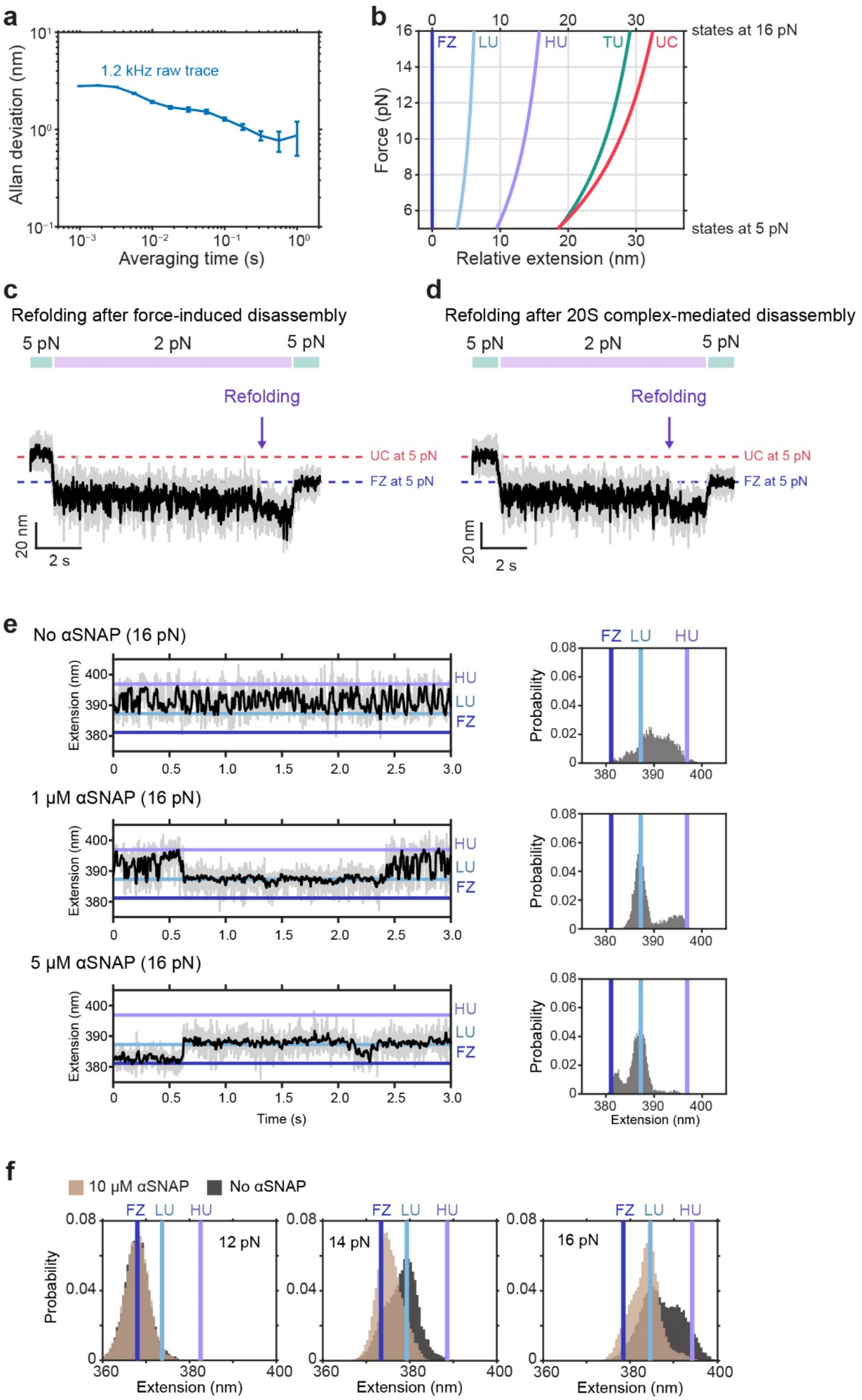
a
1.2 kHz raw trace
Allan deviation (nm)
Averaging time (s)
b
states at 16 pN
states at 5 pN
FZ
LU
HU
TU
UC
Force (pN)
Relative extension (nm)
c
Refolding after force-induced disassembly
5 pN
2 pN
5 pN
Refolding
UC at 5 pN
FZ at 5 pN
20 nm
2 s
d
Refolding after 20S complex-mediated disassembly
5 pN
2 pN
5 pN
Refolding
UC at 5 pN
FZ at 5 pN
20 nm
2 s
e
No αSNAP (16 pN)
1 μM αSNAP (16 pN)
5 μM αSNAP (16 pN)
Extension (nm)
Time (s)
HU
LU
FZ
Probability
Extension (nm)
f
10 μM αSNAP
No αSNAP
12 pN
14 pN
16 pN
FZ
LU
HU
Probability
Extension (nm)

**Supplementary Fig. 1 Modeling and monitoring extensions of SNARE complexes.**

**a** The Allan deviation of bead position as a function of averaging time for our high speed (1.2 kHz) trace. Error bars represent standard errors for 5 repeated measurements on the same construct. **b** Relative extension of the states of the SNARE complex at varying forces. The lines are the WLC model assuming a persistent length of 0.77 nm. **c**, **d** Representative refolding traces of the SNARE complex after force-induced disassembly (**c**) and 20S complex-mediated disassembly (**d**). **e** Representative trace and extension histogram of Fully-zippered, Linker-unzipped, and Half-unzipped states under 16 pN of force at different αSNAP concentrations. **f** Extension histogram of Fully-zipped, Linker-unzipped, and Half-unzipped states under different forces (12 pN, 14 pN, 16 pN) with or without αSNAP. Throughout the figure, gray and black traces are 1.2-kHz raw and 60-Hz-filtered traces, respectively. FZ: fully-zippered; LU: linker-unzipped; HU: half-unzipped; TU: totally-unzipped; UC: unstructured-coil.

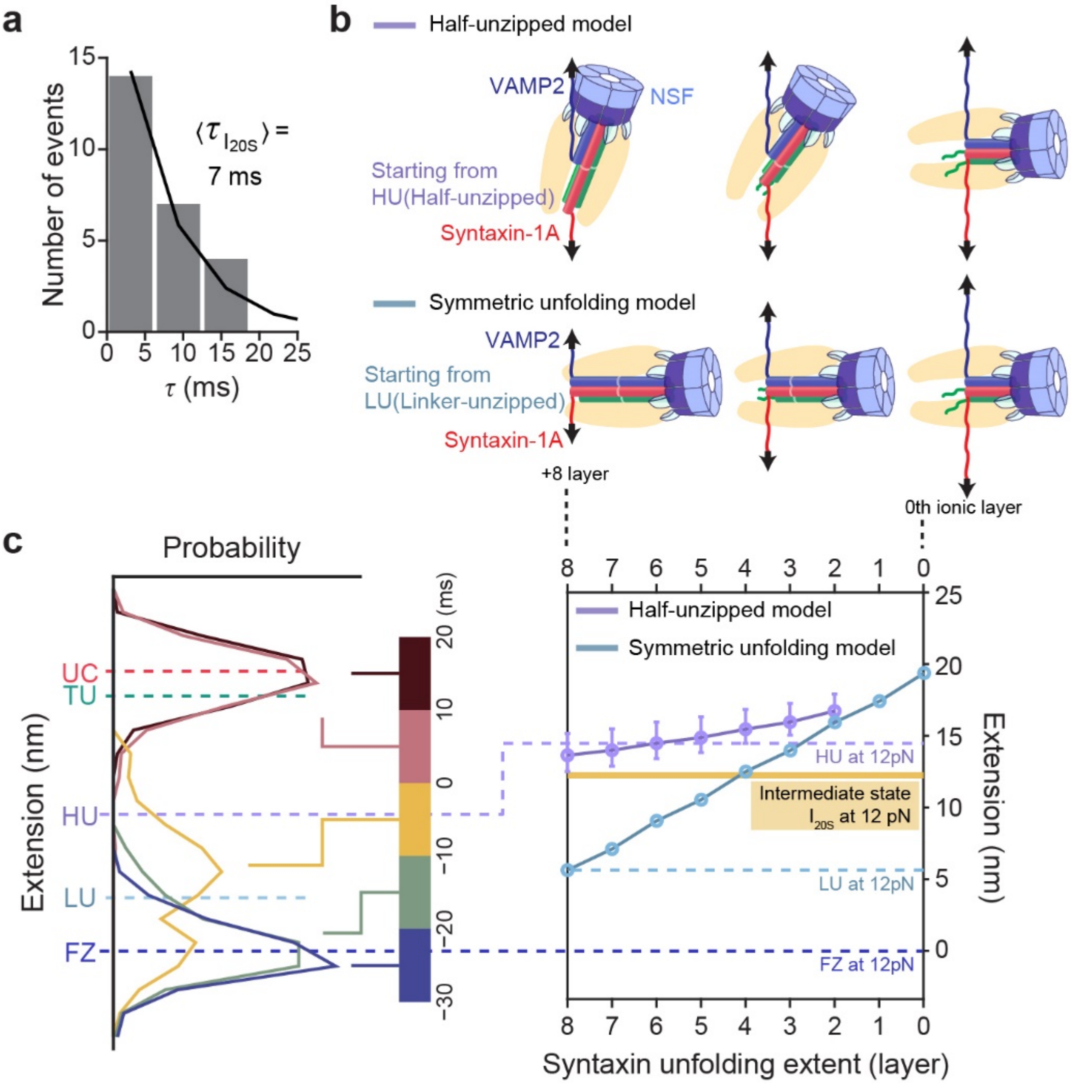


**Supplementary Fig. 2 Model of SNARE complex unzipping and characterization of the intermediate state.**

**a** Dwell time distribution of the intermediate state for the same data as in Fig. 1i ($N$ = 26). **b** Schematic model of SNARE complexes, following two different models regarding the intermediate state. The half-unzipped model starts from the HU state with unfolded VAMP2 from the beginning. The symmetric-unfolding model starts from the LU state, and VAMP2 symmetrically unfolds with syntaxin. **c** Two models (Half-unzipped, Symmetric-unfolding) plotted with the extent of syntaxin unfolding and extension level. In the half-unzipped model, the lower extent and upper extent of error bar demonstrate VAMP2 unfolding to +2 layer and to 0 layer, respectively[1]. The extension level at the intermediates (yellow line) is positioned between HU and LU and meets with the Symmetric-unfolding model plot (cyan-blue line).

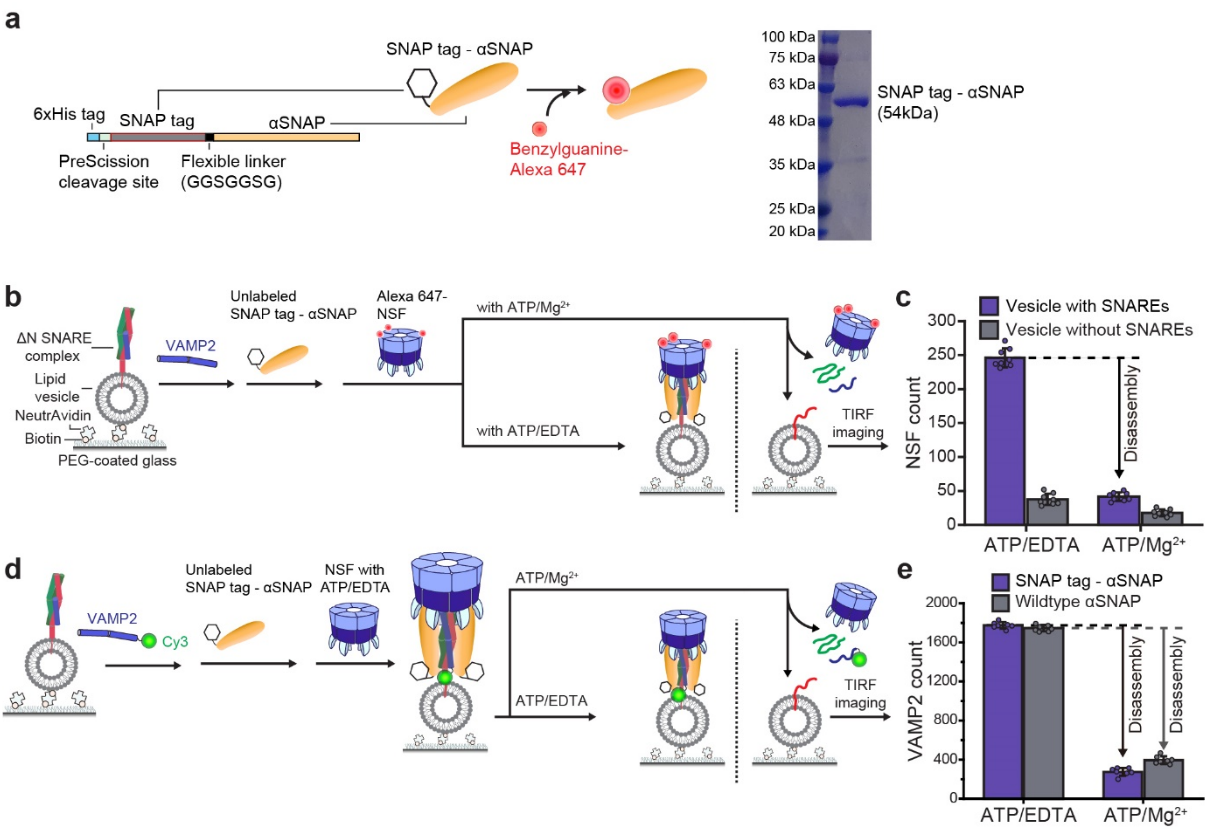


**Supplementary Fig. 3 Control experiments for SNAP tag fused αSNAP.**
**a** Design of SNAP tag-αSNAP with flexible linker and His tag/PreScission cleavage site for purification. After purification, SNAP tag was labeled with Benzylguanine(BG)-Alexa647 dye. SDS-PAGE gel image of SNAP tag-αSNAP after affinity and size exclusion chromatography. A representative gel is shown from three independent experiments. Full gels are shown in Source data file. **b** Schematic for measuring the Alexa647-labeled NSF count during NSF-mediated SNARE complex disassembly using unlabeled SNAPtag-αSNAP. The schematic shows successful trapping of the 20S complex in 1 mM ATP/1 mM EDTA and following disassembly under 1 mM ATP/10 mM Mg2+ condition. **c** The number of labeled NSF spots under the two conditions in (**b**) with SNARE reconstituted vesicles or vesicles without SNAREs as a negative control. **d** Schematic for measuring the Cy3-labeled VAMP2 count during 20S complex-mediated SNARE complex disassembly. After assembling the 20S complex with 1 mM ATP/1 mM EDTA, the complex was incubated with 1 mM ATP/1 mM EDTA or 1 mM ATP/10 mM $Mg^{2+}$ for disassembly. **e** The number of Cy3-VAMP2 spots under the two conditions described in (**d**) using SNAP tag-αSNAP and wild type αSNAP. Error bars in (**c** and **e**) represent mean ± s.d. for n=8 (**c**) and n=7 (**e**) images from 2 independent

experiments. Source data are provided as a Source Data file.

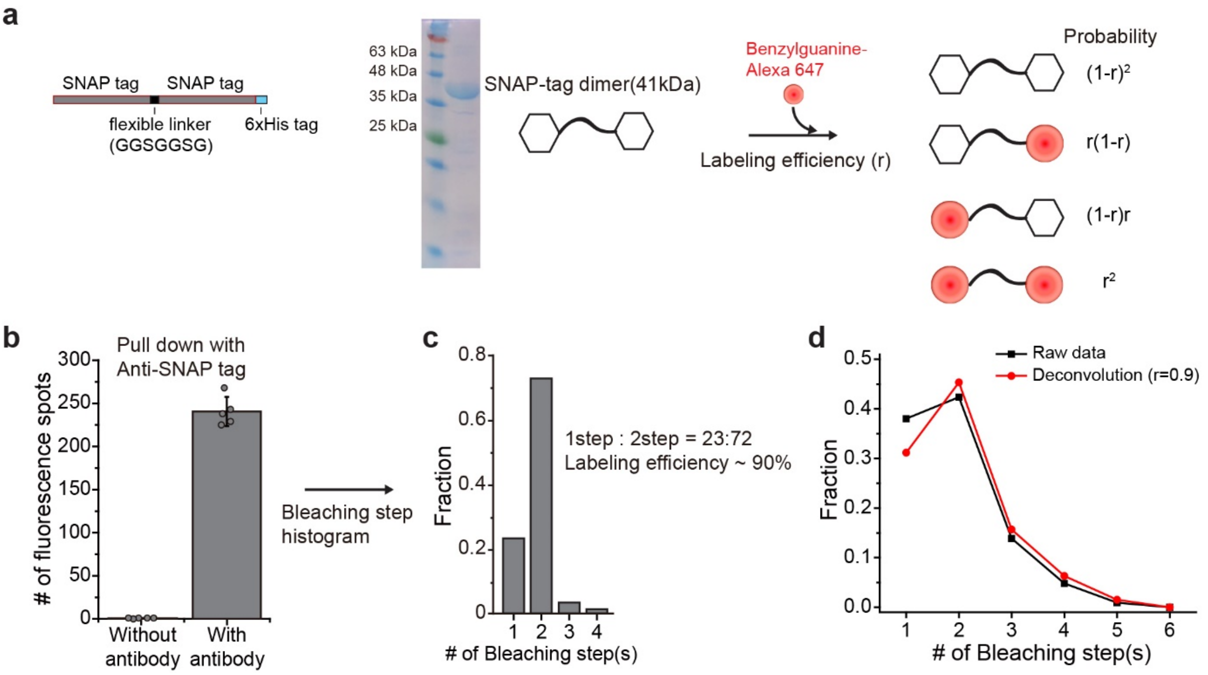


**Supplementary Fig. 4 The measurement of labeling efficiency to create deconvoluted photobleaching histograms.**

**a** The design of the SNAP tag dimer with a flexible linker and a His tag for purification. SDS-PAGE gel image of SNAP tag dimer after affinity and size exclusion chromatography. The theoretical probability was calculated for four different labeling cases where r is labeling efficiency. A representative gel is shown from two independent gel from same sample. Full gels are shown in Source data file. **b** Characterization of SNAP tag dimer using an anti-SNAP tag antibody. **c** Distribution of the photobleaching step(s) of SNAP tag dimer (N = 672 molecules), indicating a high labeling efficiency, r~0.9. **d** Example of the deconvoluted photobleaching step(s) considering 90% labeling efficiency compared to the raw data. The detail is described in method. Error bars in (**b**) represent mean ± s.d. for n=5 images. Source data are provided as a Source Data file.

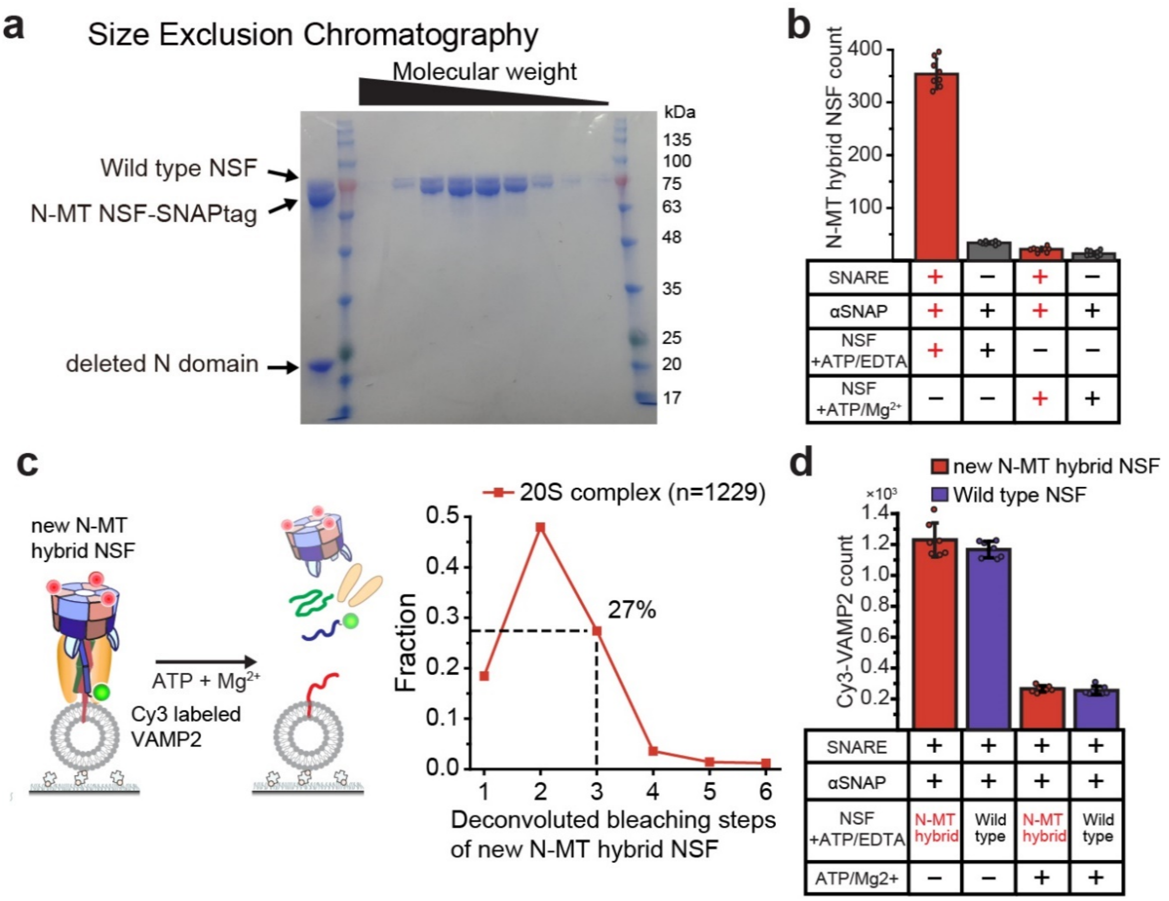


**Supplementary Fig. 5 Preparation and additional experiments for N-MT hybrid NSF.** **a** SDS-PAGE gel image of N-MT hybrid NSF after affinity and size exclusion chromatography (SEC). N domains of N-5MT NSF protomers deleted by PreScission were removed during SEC. A representative gel is shown from two independent gel from same sample. **b** Counts for N-MT hybrid NSF under ATP-non-hydrolyzing (1 mM ATP/1 mM EDTA) and hydrolyzing (1 mM ATP/10 mM $Mg^{2+}$) conditions. **c** The distributions of deconvoluted photobleaching step(s) for new N-MT hybrid NSF (N = 1229 molecules) binding to SNARE-αSNAP complex. New N-MT hybrid NSF consisted of a larger fraction of N-MT subunits than used in Fig. 3. **d** Comparison of SNARE disassembly activity of wild type NSF and new N-MT hybrid NSF while inducing ATP hydrolysis (+ATP/$Mg^{2+}$) in pre-made 20S complex. Error bars in (**b** and **d**) represent mean ± s.d. for n=7 (**b**) and n=7 (**d**) images from 2 independent experiments. Source data are provided as a Source Data file.

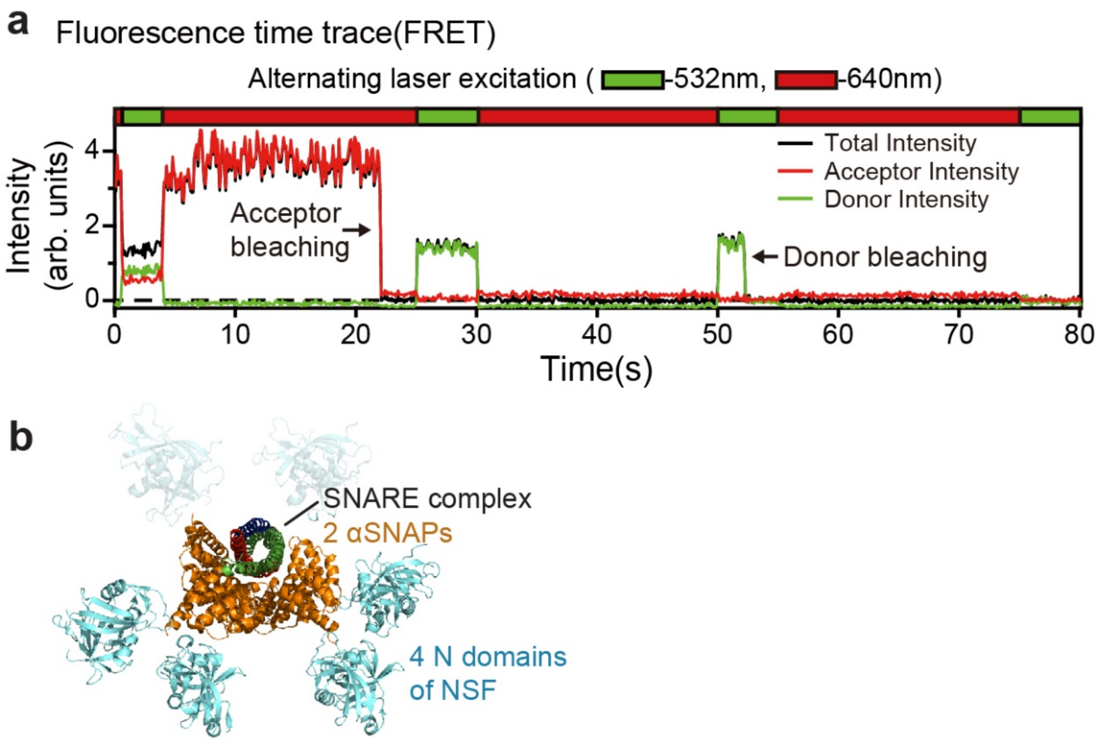


**Supplementary Fig. 6 Alternating excitation for single pair FRET experiments and structure of 20S complex.**

**a** Full representative trace of a FRET measurement. Alternating laser excitation was used to select NSF with one donor and one acceptor dye from the photobleaching step. The fluorescence time trace in Fig. 4 was created by collecting the traces only when excitation of the green (532 nm) laser. **b** The 20S complex structure containing two αSNAPs interacting with four N domains of NSF (PDB ID: 6MDM[2]). The remaining two N domains of the NSF were not included in the structure, but are marked transparently.

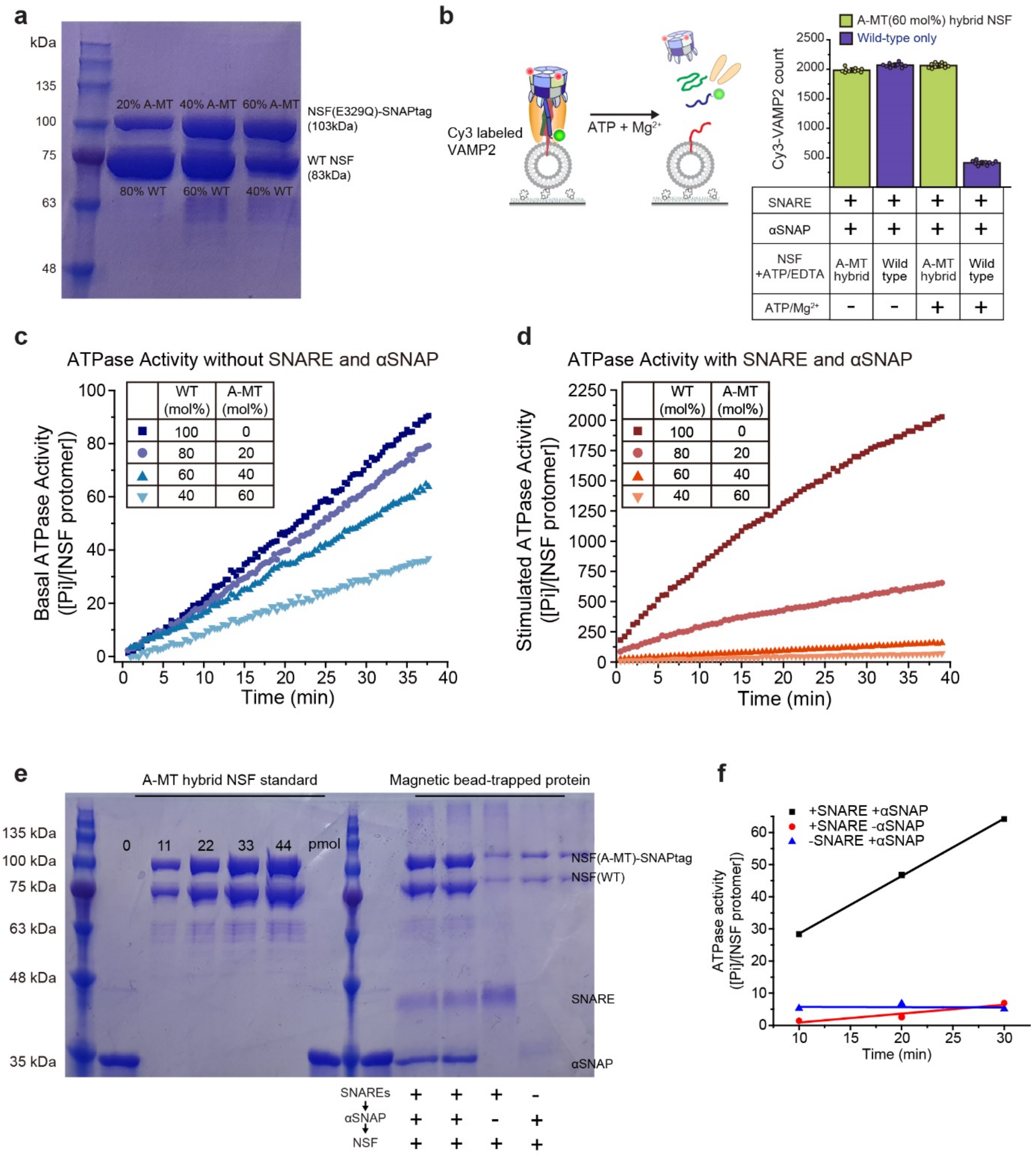


**Supplementary Fig. 7 Preparation of A-MT hybrid NSF and detailed results from the ATPase assay**.

**a** SDS-PAGE gel image of A-MT hybrid NSF at different (20%, 40%, 60%) A-MT protomer ratios. A representative gel is shown from two independent gel from same sample. Full gels are shown in Source data file. **b** Measurements of SNARE disassembly activity of A-MT (60%) hybrid NSF compared to WT NSF. 20S complex was pre-formed under non-hydrolyzing (1 mM ATP/1 mM EDTA) conditions and SNARE disassembly was induced by

buffer exchange to 1 mM ATP/10 mM $Mg^{2+}$ for 5 minutes. **c, d** Kinetic curves for free NSF ATPase activity at four different A-MT protomer ratios without (**c**) or with (**d**) SNARE (300 nM) and αSNAP (1 μM). **e** Full image of the SDS-PAGE gel in Fig. 5h. The amounts of the standard samples were measured using the Bradford assay before loading. After the ATPase activity measurements, magnetic bead-trapped protein was solubilized and loaded with SDS-PAGE loading dye. **f** Representative kinetic curves of ATPase activity for magnetic bead-trapped NSF with or without SNARE and αSNAP. Error bars in (**b**) represent mean ± s.d. for n=10 images. Source data are provided as a Source Data file.

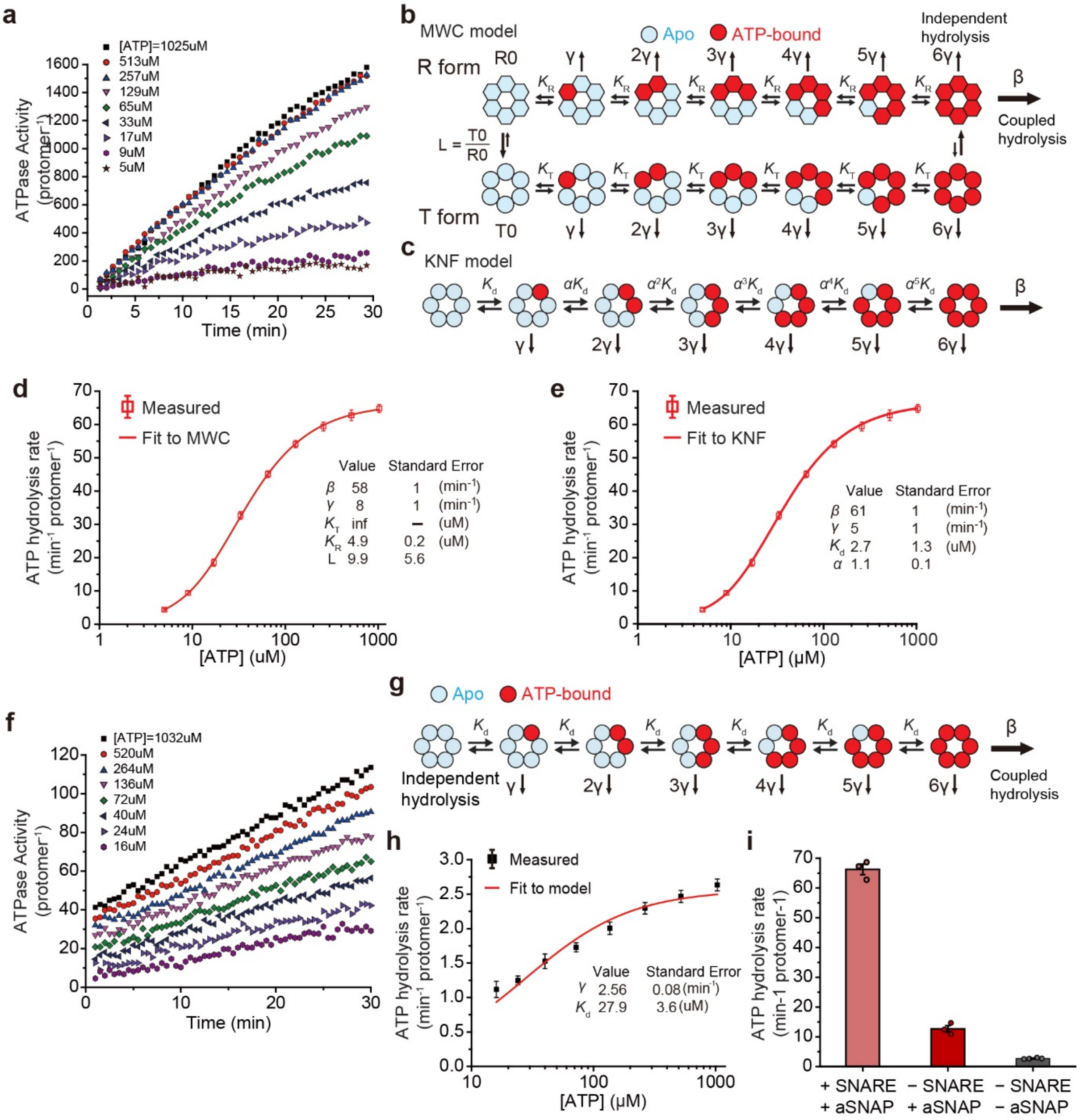

a
[ATP]=1025uM
513uM
257uM
129uM
65uM
33uM
17uM
9uM
5uM
ATPase Activity (protomer⁻¹)
Time (min)
b
MWC model
Apo
ATP-bound
Independent hydrolysis
R form
R0
T form
T0
L = T0/R0
β
Coupled hydrolysis
c
KNF model
d
Measured
Fit to MWC
Value Standard Error
β 58 1 (min⁻¹)
γ 8 1 (min⁻¹)
K_T inf — (uM)
K_R 4.9 0.2 (uM)
L 9.9 5.6
ATP hydrolysis rate (min⁻¹ protomer⁻¹)
[ATP] (uM)
e
Measured
Fit to KNF
Value Standard Error
β 61 1 (min⁻¹)
γ 5 1 (min⁻¹)
K_d 2.7 1.3 (uM)
α 1.1 0.1
ATP hydrolysis rate (min⁻¹ protomer⁻¹)
[ATP] (μM)
f
[ATP]=1032uM
520uM
264uM
136uM
72uM
40uM
24uM
16uM
ATPase Activity (protomer⁻¹)
Time (min)
g
Apo
ATP-bound
Independent hydrolysis
Coupled hydrolysis
h
Measured
Fit to model
Value Standard Error
γ 2.56 0.08 (min⁻¹)
K_d 27.9 3.6 (uM)
ATP hydrolysis rate (min⁻¹ protomer⁻¹)
[ATP] (μM)
i
ATP hydrolysis rate (min-1 protomer-1)
+ SNARE + aSNAP
− SNARE + aSNAP
− SNARE − aSNAP

**Supplementary Fig. 8: Additional fitting and experiments for NSF's ATPase activity.** **a**, **f** Representative kinetic curves of NSF's ATPase activity in the 20S complex (**a**) and free NSF (**f**) using various ATP concentrations. **b**, **c** Cooperative model for ATP binding and hydrolysis if NSF in the 20S complex based on the MWC model (**b**) and KNF model (**c**). **d** Fitting result from the model described in (**b**). The infinite value of the $K_T$ means ATP-bound NSF exists only in the R form and that this result reduces the model in (**b**) to the one-layer model described in Fig. 6a. **e** Fitting result from the model described in (**c**). Since the scaling factor $\alpha$ is nearly 1, the model is also reduced to what is described in Fig. 6a. **g** Model of ATP hydrolysis cycle for free NSF with the same dissociation constant $K_d$. **h** Fitting result from the model described in (**g**). The fit did not converge well. **i** The effect of αSNAP to the ATPase activity of NSF without SNARE complex. The +/+ and -/- data are the same data in Fig. 5. The data in (**d, e** and **h**) are the same as those in Fig. 6. The -/+ data in (**i**) is mean ± s.e.m. for 3 independent experiments. $K_T$, $K_R$, and $K_d$: dissociation constant for ATP binding in subunits; $\gamma$: independent hydrolysis rate; $\beta$: coupled hydrolysis rate; $L$: allosteric constant, that is the ratio of proteins in the T and R form in the absence of ATP. $\alpha$: scaling factor for $K_d$. Source data are provided as a Source Data file.

## Supplementary References


1. Gao, Y. et al. Single Reconstituted Neuronal SNARE Complexes Zipper in Three Distinct Stages. *Science* **337**, 1340 (2012).
2. White, K.I., Zhao, M., Choi, U.B., Pfuetzner, R.A. & Brunger, A.T. Structural principles of SNARE complex recognition by the AAA+ protein NSF. *Elife* **7**(2018).
