# Supplementary material for: Extreme parsimony in ATP consumption by 20S complexes in the global disassembly of single SNARE complexes
Source: Nat Commun. 2021 May 28;12:3206. doi: 10.1038/s41467-021-23530-0 (PMC8163800; doi:10.1038/s41467-021-23530-0)
Supplement: Supplementary file 3 — Reporting Summary [file 41467_2021_23530_MOESM3_ESM.pdf]

nature research

Corresponding author(s): Tae-Young Yoon

Last updated by author(s): Apr 12, 2021

Reporting Summary

Nature Research wishes to improve the reproducibility of the work that we publish. This form provides structure for consistency and transparency in reporting. For further information on Nature Research policies, see our Editorial Policies and the Editorial Policy Checklist.

Statistics

For all statistical analyses, confirm that the following items are present in the figure legend, table legend, main text, or Methods section.

n/a	Confirmed	
☐	☒	The exact sample size (n) for each experimental group/condition, given as a discrete number and unit of measurement
☐	☒	A statement on whether measurements were taken from distinct samples or whether the same sample was measured repeatedly
☐	☒	The statistical test(s) used AND whether they are one- or two-sided<br>*Only common tests should be described solely by name; describe more complex techniques in the Methods section.*
☒	☐	A description of all covariates tested
☒	☐	A description of any assumptions or corrections, such as tests of normality and adjustment for multiple comparisons
☐	☒	A full description of the statistical parameters including central tendency (e.g. means) or other basic estimates (e.g. regression coefficient) AND variation (e.g. standard deviation) or associated estimates of uncertainty (e.g. confidence intervals)
☒	☐	For null hypothesis testing, the test statistic (e.g. F, t, r) with confidence intervals, effect sizes, degrees of freedom and P value noted<br>*Give P values as exact values whenever suitable.*
☒	☐	For Bayesian analysis, information on the choice of priors and Markov chain Monte Carlo settings
☒	☐	For hierarchical and complex designs, identification of the appropriate level for tests and full reporting of outcomes
☒	☐	Estimates of effect sizes (e.g. Cohen's d, Pearson's r), indicating how they were calculated

Our web collection on statistics for biologists contains articles on many of the points above.

Software and code

Policy information about availability of computer code

Data collection	Data were collected using custom software written in LabVIEW (2015) and MATLAB (R2016b).
Data analysis	OriginPro 2015 was used for fitting curves. The custom LabVIEW and MATLAB codes used for data analysis are available at https://github.com/tyyoonlab for the magnetic tweezer experiments; https://github.com/kahutia/SingleMoleculeImageAnalyzer/releases/tag/V8.0 and https://github.com/kahutia/smFRET_trace_viewer/releases/tag/v2.1 for the fluorescence experiments

For manuscripts utilizing custom algorithms or software that are central to the research but not yet described in published literature, software must be made available to editors and reviewers. We strongly encourage code deposition in a community repository (e.g. GitHub). See the Nature Research guidelines for submitting code & software for further information.

Data

Policy information about availability of data

All manuscripts must include a data availability statement. This statement should provide the following information, where applicable:

- Accession codes, unique identifiers, or web links for publicly available datasets
- A list of figures that have associated raw data
- A description of any restrictions on data availability

The data that support the findings of this study are available from the corresponding author upon reasonable request.

Field-specific reporting

Please select the one below that is the best fit for your research. If you are not sure, read the appropriate sections before making your selection.

- [x] Life sciences
- [ ] Behavioural & social sciences
- [ ] Ecological, evolutionary & environmental sciences

For a reference copy of the document with all sections, see nature.com/documents/nr-reporting-summary-flat.pdf

Life sciences study design

All studies must disclose on these points even when the disclosure is negative.

Sample size	The sample size for each experiment counting the number of molecules without further analysis is indicated in the figure legend. Each bleaching histogram was constructed from photo-bleaching steps measured from >1,000 molecules in more than 3 movies. For FRET analysis, traces were collected continuously until the data set was large enough to analyze the distribution.
Data exclusions	In bleaching histograms of aSNAP and NSF, data with more than 4 (aSNAP) and 6 (NSF) photo-bleaching steps, respectively, were excluded based on the structural information.
Replication	Experiments to perform further analysis were repeated independently at least three times, and experiments counting the number of molecules without further analysis were performed in duplicate. Replication was successful for all experiments.
Randomization	Randomization was not performed because of the unbiased nature of the in vitro experiments performed in this study.
Blinding	Blinding was not performed because of the unbiased nature of the in vitro experiments performed in this study.

Reporting for specific materials, systems and methods

We require information from authors about some types of materials, experimental systems and methods used in many studies. Here, indicate whether each material, system or method listed is relevant to your study. If you are not sure if a list item applies to your research, read the appropriate section before selecting a response.

Materials & experimental systems

n/a	Involved in the study	
[ ]	[x]	Antibodies
[x]	[ ]	Eukaryotic cell lines
[x]	[ ]	Palaeontology and archaeology
[x]	[ ]	Animals and other organisms
[x]	[ ]	Human research participants
[x]	[ ]	Clinical data
[x]	[ ]	Dual use research of concern

Methods

n/a	Involved in the study	
[x]	[ ]	ChIP-seq
[x]	[ ]	Flow cytometry
[x]	[ ]	MRI-based neuroimaging

Antibodies

Antibodies used	Experimental conditions including the concentrations of materials used in this study is mentioned in the Methods section.<br>Rabbit polyclonal Anti-SNAP tag antibody, called Anti-SNAP tag in this manuscript, is purchased from New England Biolabs, Cas#P9310S ;<br>Mouse monoclonal Anti-NSF antibody (C-5), called Anti-N domain in this manuscript, is purchased from Santa Cruz Biotechnology, Cas# Sc-74457;<br>Biotin-SP-AffiniPure Goat Anti-Rabbit IgG (H+L) antibody is purchased from Jackson ImmunoResearch Labs, Cat# 111-065-003;<br>Biotin-SP-AffiniPure Goat Anti-Mouse IgG (H+L) antibody is purchased Jackson ImmunoResearch Labs, Cat# 115-065-166
Validation	All antibodies are commercially validated and previously published. All information about antibodies can be found on the manufacturer's website.
